# Primary teachers’ attitudes towards using new technology and stimulating higher-order thinking in students: A profile analysis

**DOI:** 10.1007/s10639-022-11413-w

**Published:** 2022-11-11

**Authors:** Frances Wijnen, Juliette Walma van der Molen, Joke Voogt

**Affiliations:** 1 Department of Teacher Development, ELAN, University of Twente, 7522 NB Enschede, The Netherlands; 2 Faculty of Social and Behavioural Sciences, University of Amsterdam, 1012 WX Amsterdam, The Netherlands

**Keywords:** Primary school teacher, Attitude, New technology, Higher-order thinking, Cluster-analysis

## Abstract

Critical thinking, creative thinking, problem solving and other so-called higher-order thinking skills are regarded as crucial for students to develop. Research shows that technology can be used as a tool to stimulate students’ higher-order thinking skills. However, most teachers rarely use new technology to stimulate students to engage in higher-order thinking. To help teachers in this, we need to gain an understanding of teachers’ *attitudes* towards using new technology and towards stimulating higher-order thinking. In this study, we explore these teacher attitudes by identifying teacher profiles based on primary school teachers’ attitudes (N = 659) towards (a) using new technology and (b) stimulating higher-order thinking. Results of the cluster-analysis revealed three teacher profiles. In follow-up focus group interviews with 21 participants, we found that teachers recognized the identified profiles and that the results of the cluster-analysis matched teachers’ self-chosen profiles in almost all cases. These results indicate that we can suitably characterize teachers based on their attitudes towards using new technology and stimulating higher-order thinking. Identification of these profiles may help us understand why certain groups of teachers may use new technology to stimulate students’ higher-order thinking, while other teachers might not. This might provide starting points for tailored teacher professionalization for different groups of teachers.

## Introduction

Critical thinking, creative thinking, problem solving and other so-called higher-order thinking skills are regarded as very important for students to develop (Conklin 2012; Driana & Ernawati 2019). Students may actively construct knowledge and become involved in meaningful learning when they engage in higher-order thinking (Anderson et al. 2001). Researchers argue that explicit teaching of higher-order thinking is necessary, since students may not become good thinkers without support (Elder 2003; Schulz & FitzPatrick 2016). Thus, teachers need to offer experiences to students that challenge them to engage in complex cognitive skills (e.g., analyzing, evaluating, creating) (e.g., Wijnen et al. 2021a; King et al. 1998).

Higher-order thinking can be stimulated in different ways. For example, by answering teacher and/or student generated questions, reflecting on dilemmas and coming up with self-generated solutions for a problem. Furthermore, letting students work together in small groups and stimulating activities, such as group discussion, peer tutoring, and cooperative learning, are effective methods for engaging students in higher-order thinking (King et al. 1998; Singh et al. 2018).

Research shows that technology can be used as a tool to support students’ learning, including stimulating their higher-order thinking skills (Backfish et al. 2020; Mayer 2019). Teaching with technology allows for making use of the technologies’ ‘affordances’, which provides opportunities to enrich the learning environment (Ottenbreit-Leftwich et al. 2018). For example, by enhancing students’ (online) collaborative skills (e.g., via social media), or by simulating authentic problems or (aspects of) the physical world (e.g., via games augmented, virtual reality) in which skills, such as exploring, planning, designing, and creating solutions, might be practiced (Araiza-Alba et al. 2021; Chiang et al. 2014; Dede 2000; Tangkui & Keong 2020). *New* technology, such as augmented reality, virtual reality, and games have been found to advance students’ higher-order thinking, compared to teaching methods that do not include such technologies (Araiza-Alba et al. 2021; Chiang et al. 2014; Passig et al. 2016; Tangkui & Keong 2020). In Sect. 1.2.2. we explain how we understand new technology in this study.

Most teachers make little use of *new* technology to support students’ learning (Fraillon et al. 2018; Ottenbreit-Leftwich et al. 2018) and rarely use technology to stimulate students’ higher-order thinking (Backfish et al. 2020; Fraillon et al. 2013; Voogt et al. 2016). To better understand why teachers make little use of new technology to stimulate higher-order thinking, we need to gain an insight into teachers’ *attitudes* towards using new technology and towards stimulating higher-order thinking. The importance of attitude has been emphasized in many studies (e.g., Howe & Krosncik, 2017; Vögel & Wänke, 2016). Attitude impacts a person’s intention and behavior and how a person processes information (Vögel & Wänke, 2016). Furthermore, attitude-based professionalization has been found to have a positive impact on teachers’ teaching behavior in other contexts such as science education (Van Aalderen-Smeets & Walma van der Molen 2015). More specifically, research shows that teachers’ attitudes towards using technology in teaching impacts teachers’ technology use (e.g., Bowman et al. 2020; Farjon et al. 2019). Similarly, teachers’ attitudes towards stimulating higher-order thinking impact teachers’ teaching practices (Wijnen et al. 2021b).

To support teachers in their use of new technology to stimulate students’ higher-order thinking, it is important to gain insight into teachers’ attitudes towards using new technology *and* towards stimulating higher-order thinking. However, a priori we do not assume a fixed relationship between these two attitudes. We expect that teachers may differ in their attitudes towards stimulating students’ higher-order thinking and using new technology. For example, a teacher may have a positive attitude towards stimulating students’ higher-order thinking, and a negative attitude towards using new technology, whereas another teacher may have a positive attitude towards both teaching approaches. To explore which ‘combinations of attitudes’ exist, we aimed to identify teacher profiles. This means that we combined measurements of teachers’ attitudes towards stimulating students’ higher-order thinking and towards using new technology and evaluated to what extent participants scored similarly or differently on these measurements.

### Aim of the study

Based on the considerations above, we aimed to answer two research questions in this study: (a) which teacher profiles can be identified, based on teachers’ attitudes towards using new technology in teaching and towards stimulating higher-order thinking in students? and (b) do teachers recognize the identified profiles?

For the identification of teacher profiles, we measured primary school teachers’ attitudes towards (1) stimulating higher-order thinking in students and (2) using new technology in teaching with two separate surveys. We see two important reasons for measuring these attitudes separately. First, as described above, teachers may differ in their attitudes towards using new technology and stimulating higher-order thinking (Cheeseman 2018; Önal et al. 2017; Prieto et al., 2016; Schulz & FitzPatrick 2016). Second, research shows that these attitudes are made up of different attitudinal factors (Wijnen et al. 2021a). For example, in the context of stimulating higher-order thinking, studies indicate that teachers may believe that stimulating higher-order thinking may not be suitable for low-achieving students (e.g., Zohar et al. 2001), whereas in the context of technology use such beliefs did not come up in research.

After measuring these attitudes separately, we conducted a cluster analysis to identify teacher profiles. Identifying teacher profiles based on teachers’ views and use of *technology* is not new. In 2017, Admiraal et al., identified five types of teachers based on their beliefs regarding teaching and technology use. In 2021, Howard et al., identified teacher profiles based on teachers’ perceptions regarding their readiness to transition to online teaching. In 2022, Admiraal identified teacher profiles based on teachers’ use of Open Educational Resources (OER). To our knowledge, however, there are no studies in which teacher profiles are identified based on teachers’ attitudes towards stimulating higher-order thinking in students with or without the use of technology. We believe that identification of teacher profiles is valuable because this could provide insight in the needs for teacher support for different groups of teachers, which may allow us to develop teacher-tailored professionalization that fit these needs. Furthermore, identification of such profiles could give us a better understanding of how different teachers view the use of new technology and of stimulating higher-order thinking in students and to what extent this impacts their teaching behavior. This might help us understand, whether, how and why teachers use new technology and/or stimulate students’ higher-order thinking.” Furthermore, the above-mentioned studies were conducted with secondary school teachers, while our study focuses on primary education.

### Definitions

#### Stimulating higher-order thinking

Anderson et al. (2001) revised Bloom’s well know taxonomy of cognitive processes. They distinguished between lower-order thinking skills (remembering, understanding and applying) and higher-order thinking skills (analyzing, evaluating, and creating). King et al., (1998) state that successful application of higher-order thinking, like critical, reflective and creative thinking, should result in some outcome (e.g., a decision, explanation, or product). Furthermore, engaging in higher-order thinking fosters the development of these thinking skills in students (King et al. 1998).

To define *stimulating* higher-order thinking, since we focus on the role of the teacher, we used the descriptions of King et al., (1998) and Bloom’s revised taxonomy (Anderson et al. 2001). This resulted in the following definition: “stimulating higher-order thinking in students means offering assignments, questions, problems or dilemmas where students need to use complex cognitive skills (such as analyzing, evaluating and creating) in order to find a solution or make a decision, prediction, judgement or product” (cf. Wijnen et al. 2021a, p. 5).

#### New technology

The term *new* technology is difficult to define because whether something is new, differs between people and contexts and may therefore have a different meaning for different people. Therefore, it is important to take the teachers’ perspective and context into account. What is ‘new’ is dependent upon what technology is available for teachers to use (context), whether and how a teacher believes that a specific technology can be used to support students learning, and whether a teacher is aware of the affordances that specific technologies offer.

Although teachers use different forms of technology in their teaching, such as computers and digital whiteboards (Smeets & Van der Horst, 2018), the implementation of ‘new’ technologies such as virtual reality, educational robots, and 3D-printers is still not very common (Fraillon et al., 2018) even though these technologies provide opportunities to enhance students’ learning (Backfish et al., 2020). These results might indicate that many teachers are unaware of the opportunities that various technologies provide to enrich the learning environment, or that they are unsure how such technologies can be used to enhance student learning.

To define ‘new technology’ in this study, we decided to focus on the use of technology to support students’ learning. Furthermore, we provided examples of technologies that are not often used by (Dutch) teachers (Smeets, 2020; Voogt et al., 2016). These studies show that teachers mainly use the interactive whiteboard to support their instruction and hardly use other technologies (e.g., robots, virtual reality) to enhance their teaching practices. This resulted in the following description: “New technology refers to hardware and software that teachers can use to support and/or enrich their teaching practices. Some examples of hardware are: smartphones, tablets, 3D printers and educational robots (BeeBot, DASH). Software examples are: simulation software, design software, programming software and video-editing software.” (Wijnen et al. 2021a, p. 4). This description was presented to primary school teachers to evaluate to what extent it fits teachers’ perception of the term new technology.

#### Attitude

According to Ajzen (2001), “attitude represents a summary evaluation of a psychological object (the ‘attitude-object’), captured in such attribute dimensions as good-bad, harmful-beneficial, pleasant-unpleasant, and likeable-dislikeable” (p. 28). An attitude-object is the entity about which an attitudinal evaluation is made (Ajzen, 1991, 2001). In this study, there are two attitude-objects, namely ‘using new technology in teaching’ and ‘stimulating higher-order thinking in students.’

Based on the Theory of Planned Behavior (TPB; Ajzen, 1991, 2001), we view attitude as an umbrella term that consists of three dimensions. These dimensions can be comprised of subcomponents, which together form a person’s attitude towards a specific behavior (cf. Wijnen et al. 2021a). The *perceptions of behavioral attributes* dimension refers to beliefs and feelings a person may associate with a specific behavior, in this case, using new technology in teaching and stimulating students’ higher-order thinking, respectively. The *perceptions of social norms* dimension refers to a person’s perceived social acceptability of the behavior. The *perceptions of behavioral control* dimension refers to a person’s perceived level of control he/she has in performing the behavior. These perceptions can refer to external factors (e.g., availability of time or resources) or internal factors (e.g., perceived capability of performing the behavior, often described as “self-efficacy”, based on Bandura’s concept) (Ajzen, 2002; Armitage & Conner, 2001).

A person’s attitude can influence a person’s intention to engage in a specific behavior, which is assumed to impact actual behavior (Ajzen, 1991). To explore the relationship between the different attitudinal profiles and teacher behavior, we therefore also included measures of teachers self-reported behavior related to new technology use and to stimulating higher-order thinking in their students.

## Method

### Data collection and analysis

For this study, data was collected in two stages. In the first stage, two questionnaires were administered to a large group of primary school teachers in the Netherlands. With this data, we conducted a cluster-analysis to identify teacher profiles based on their attitudes towards (1) using new technology in teaching, and (2) stimulating higher-order thinking. In the second stage, we conducted follow-up focus group interviews with several teachers who also completed the questionnaires.

### Stage 1: Identifying teacher profiles

#### Participants and procedure

Two questionnaires (see 2.2.2.) were administered simultaneously to a large group of third- and fourth-year pre-service (*N* = 257) and in-service primary school teachers (*N* = 402) in the Netherlands. The effective sample size consisted of 136 (20.6%) males and 523 (79.4%) females. Participants’ age ranged from 18 to 65 years old (*M* = 29.52, *SD* = 12.69)*.*

Primary schools (in-service teachers) and teacher education colleges (pre-service teachers) were visited by one of the researchers. After a brief introduction and giving informed consent, teachers were directed to an online (84.2%) or paper-and-pencil version (15.8%) of the questionnaires. It took participants approximately 25 min to complete both questionnaires. In a few cases, having the researcher visit the school was not possible. Therefore, a small number of participants received an email with a link to redirect them to the online version of the questionnaires.

#### Instruments

##### The TANT (Teachers’ Attitudes towards New Technology) questionnaire

The TANT questionnaire measures (pre- and in-service) primary school teachers’ attitudes towards using new technology in teaching. The TANT questionnaire meets the requirements for construct validity and measurement invariance (Wijnen et al. 2022).

This questionnaire consists of six scales representing six attitudinal factors. Table 1 provides an overview of these scales. The items are measured with a 5-point Likert scale ranging from (1) strongly disagree to (5) strongly agree. In addition, the TANT questionnaires includes a scale on self-reported new technology use (Composite Reliability = 0.81) which consists of seven items such as: How often do students use new technology to work on challenging problems (such as designing gymnastics gear) in your lessons? These items were measured with a 7-point Likert scale ranging from (1) never to (7) every day.

**Table 1:** Scales and items of the TANT questionnaire

Scale	Description	Example item	Number of items	Composite reliability
Perceived Relevance	Refers to teachers’ beliefs about the importance of using new technology in their teaching in order to prepare learners for later life	I think it is very important for the future of learners that they get the opportunity to learn how to work with new technology at school	3	0.77
Perceived Usefulness	Refers to teachers’ beliefs about the usefulness of new technology for improving and/or enriching their teaching and the learning of their students	I think that, with the help of new technology, I can vary more in the assignments I offer my learners	2	0.70
Perceived difficulty	Refers to teachers’ beliefs and related feelings of anxiety about the difficulty of using new technology in teaching	I think it is very difficult to use new technology in my lessons	6	0.86
Self-efficacy	Refers to teachers’ self-perceived capability to use new technology in their teaching	I am well able to choose new technologies that support the lesson content of the subjects I teach	6	0.86
Context-dependency	Refers to teachers’ *perceptions* that external factors, such as the availability of technical resources, on-site support, and available time, are *a prerequisite* for them to be able to use new technology	For me, the availability of content support, in the form of an ICT-coordinator, determines whether I use new technology in my lessons	3	0.72
Subjective Norm	Refers to teachers’ perceptions as to whether other people who are important to that teacher think it is good or bad to use new technology in teaching	I have the feeling that using new technology in lessons is appreciated by colleagues and management at our school	4	0.77

It is important to note, that the behavioral scale is *not* included in our cluster-analysis for the identification of teacher profiles. We aim to identify teacher profiles based on teachers’ attitudes, so only the attitudinal factors were included. However, we do include the behavioral scale in our post-hoc analysis to evaluate possible differences in new technology use by teachers with different profiles.

##### The SHOT (Stimulating Higher-Order Thinking) questionnaire

The SHOT questionnaire measures (pre- and in-service) primary school teachers’ attitudes towards stimulating higher-order thinking in students. The SHOT questionnaire meets the requirements for construct validity and measurement invariance (Wijnen et al. 2021b).

This questionnaire consists of four scales, which represent four attitudinal factors. Table 2 provides an overview of these scales. The items are measured with a 5-point Likert scale ranging from (1) strongly disagree to (5) strongly agree. In addition, the SHOT questionnaire contains two scales to measure teachers’ self-reported behavior related to stimulating higher-order thinking. Teacher Activities (TA; 3 items, Composite Reliability = 0.91) refers to different activities a teacher can undertake to stimulate higher-order thinking (e.g., design a lesson, teach a lesson, give assignments). Encouraging Students (ES; 2 items, Composite Reliability = 0.88) refers to activities to encourage students to engage in different complex thinking processes (e.g., problem solving, creating new products). These behavioral scales were measured with a 7-point Likert scale ranging from (1) never to (7) every day.

**Table 2:** Scales and items of the SHOT questionnaire

Scale	Description	Example item	Number of items	Composite reliability
Perceived Relevance	Refers to teachers’ beliefs about the importance of stimulating higher-order thinking for students’ personal development	I think it is essential for the learning of students that they are encouraged to engage in higher-order thinking	4	0.90
Perceived Student Ability	Refers to teachers’ beliefs about whether higher-order thinking is suitable for both low- and high-achieving students	I think that assignments that require higher-order thinking are more appropriate for 'smart' students than for 'weak' students	6	0.81
Self-efficacy	Refers to teachers’ self-perceived capability to stimulate higher-order thinking in students	I am well able to guide students in doing assignments that stimulate them to engage in higher-order thinking	4	0.90
Context-dependency	Refers to teachers’ perception that external factors, such as available time, or support are a *prerequisite* for them to be able to stimulate higher-order thinking in students	For me, making higher-order thinking assignments is only possible when I have a method that describes how to do that	4	0.73

Similarly as for the TANT questionnaire, we only included the attitudinal factors in our cluster analysis for the identification of teacher profiles. The behavioral scales were only included for the post-hoc analysis to evaluate possible differences in teaching practices aimed at stimulating students’ higher-order thinking between teachers with different profiles.

#### Analyses

To identify teacher profiles, we evaluated to what extent participants scored similarly or differently on the scales of the TANT and SHOT questionnaires, by conducting a cluster-analysis. For this analysis we used the unweighted average scores of the six attitudinal scales of the TANT questionnaire and the unweighted average scores of the four attitudinal scales of the SHOT questionnaire. We *did not* include the behavioral scales for the identification of teacher profiles. We conducted a hierarchical and follow-up k-means cluster analysis, using SPSS version 24.0.

To identify how many potential clusters there were in the data, we conducted a hierarchical cluster analysis using Ward’s linkage and the squared Euclidian distance measure (Allen & Goldstein, 2013; Sarstedt & Mooi, 2019). Next, we calculated the Variance Ratio Criterion (VRC; ﻿Calinski & Harabasz, 1974) to explore which number of clusters best fit our data. The number of clusters that maximizes the VRC indicates the appropriate number of clusters. Since VRC usually decreases with an increasing number of clusters, we also calculated the ω which refers to the relative loss of variance explained by using less clusters. Therefore, the most optimal number of clusters has the highest VRC and the lowest ω (Calinski & Harabasz, 1974).

After determining the optimal number of clusters, we conducted a k-means cluster analysis to identify groups of participants that score similarly on the TANT and SHOT questionnaire. To determine the stability of the cluster solution, the k-means analysis is performed on an approximately 50% random sample of the 659 participants and the outcome of this analysis is compared to the outcome of the k-means analysis on the full sample. Furthermore, k-means was performed multiple times where the ordering of objects (e.g., participants scores) was varied to evaluate whether the resulting clusters remained similar, which is an indication of cluster stability. Lastly, we performed a One-way ANOVA to explore differences between the clusters, which allowed us to identify different ‘types’ of teachers.

### Stage 2: Understanding teacher profiles

#### Participants and procedure

After identifying teacher profiles, we conducted five follow-up focus group interviews with 14 in-service and 7 pre-service primary school teachers. For the selection of participants, we evaluated at what schools or teacher education colleges a specific profile was strongly represented. This means that, for example, teachers from the school with the highest percentage of participating teachers that, according to our cluster analysis, belong to profile 1, were invited to participate in our focus group interviews. We did this, for each of the three clusters. This way we aimed to ensure a representation of teachers from all profiles in our focus group interviews.

Due to the outbreak of the Covid-19 pandemic, only the first focus group interview took place in a face-to-face setting. The other focus group interviews were conducted digitally with Microsoft Teams. The interviews were audio recorded. The procedure was similar in both settings. After giving informed consent, the interviewer gave an introduction about the procedure of the interview. Next participants received a description of the three identified profiles (Appendix 1) and were asked to fill in a short open-ended questionnaire about these profiles (Appendix 2). Then the semi-structured interview started. These interviews took approximately 25 min.

#### Analyses

To analyze the interview data, we created an overview of the participants’ answers of the questionnaire and transcribed all recordings of the focus group interview. With this data we aimed to answer the question: to what extent do participants recognize themselves in the profiles that were identified with the cluster analysis? In addition, we evaluated whether the teacher’s own choice of profile matched the results from the cluster analysis. To do this, we requested that participants made a code for both the TANT- and SHOT-questionnaire as well as the open-ended questionnaire that was used in the interviews. This way, we were able to compare the results from the cluster-analysis with the teachers’ own chosen profiles.

## Results

### Stage 1: Identifying teacher profiles

**Fig. 1:**
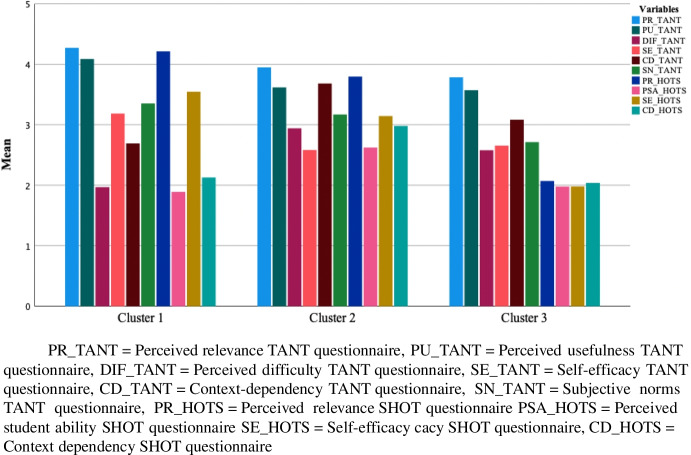
Bar chart representing the final cluster solution

Inspection of the resulting dendrogram of the hierarchical cluster analysis, indicated that a three-cluster solution might be suitable. In addition, the VRC score was the highest, VRC = 1556.945 and ω the lowest, ω = -217.023 for the three-cluster solution, indicating that three is the most optimal number of clusters for our dataset. Results of the comparison of 50% of the sample with the full sample showed that the maximum relative difference in cluster size is 5.4%, which is below the threshold of 20% (Sarstedt & Mooi, 2019). Furthermore, the multiple k-means analyses where the ordering of objects was varied, showed that the variation between the different cluster-solutions was less than 0.2%, indicating a very stable cluster solution. Figure 1 represents the final k-means cluster solution.

To identify different types of teachers, we conducted a One-way ANOVA, using the cluster number (e.g., to which cluster the participant belongs) as the grouping variable. For this analysis we included the behavioral factors (new technology use and stimulating higher-order thinking: teaching activities and encouraging students). The One-way ANOVA showed significant differences between the clusters on all variables. A Bonferroni post-hoc analysis was conducted to explore on which variables there were differences between the clusters (see Table 3). Based on these scores we distinguished three profiles of teachers.

**Table 3:** Average mean scores on the attitudinal and behavioral scales per cluster

	Profile 1		Profile 2		Profile 3		Total	
	N = 197		N = 330		N = 131		N = 658	
	*M*	*SD*	*M*	*SD*	*M*	*SD*	*M*	*SD*
TANT questionnaire								
Attitudinal scales								
Perceived relevance	**4.27**	0.555	3.95	0.660	3.79	0.760	4.01	0.676
Perceived usefulness	**4.09**	0.711	3.62	0.743	3.57	0.724	3.75	0.762
Perceived difficulty	1.97	0.632	**2.94**	0.655	2.58	0.716	2.58	0.783
Self-efficacy	**3.19**	0.709	2.58	0.614	2.65	0.639	2.78	0.701
Context dependency	2.69	0.891	**3.68**	0.730	3.08	0.795	3.27	0.907
Subjective norms	**3.35**	0.771	3.17	0.704	2.71	0.779	3.13	0.772
Behavioral scale								
New technology use^a^	**2.42**	1.043	2.02	0.921	1.92	0.464	2.12	0.912
SHOT questionnaire								
Attitudinal scales								
Perceived relevance	**4.22**	0.591	3.80	0.587	2.07	0.491	3.58	0.961
Perceived student ability	1.89	0.617	**2.62**	0.738	1.98	0.416	2.27	0.737
Self-efficacy	**3.55**	0.652	3.14	0.619	1.98	0.409	3.03	0.811
Context dependency	2.13	0.651	**2.98**	0.600	2.04	0.459	2.54	0.738
Behavioral scales								
Stimulating HOT, TA^a^	**3.48**	1.475	2.84	1.313	2.01	0.590	2.86	1.357
Stimulating HOT, ES^a^	**4.52**	1.576	4.13	1.622	2.25	1.084	3.87	1.725

**Bold**: significantly higher score on the scale compared to other profiles

Underlined: significantly lower score on the scale compared to other profiles

TA = Teaching Activities, ES = Encouraging Students

^a.^ Measured on a 7-point Likert scale: 1. Never, 2. A few times a year, 3. Once a month, 4. A few times a month, 5. Once a week, 6. A few times a week, 7. Every day

#### Profile 1

Teachers in profile 1 believe that using new technology is very important, and that new technology is a useful tool to enrich their teaching. Furthermore, these teachers do not think that using new technology is difficult and feel reasonably capable in using new technology. These teachers feel somewhat dependent upon context factors such as technical support and feel somewhat supported by their colleagues and the school director. These teachers use new technology approximately a few times a year to once a month.

Furthermore, teachers in profile 1 believe that is important to stimulate higher-order thinking. These teachers think that higher-order thinking is appropriate for both ‘smart’ and ‘weak’ students, feel capable in stimulating higher-order thinking and do not feel dependent upon context factors such as a method to be able to stimulate higher-order thinking. These teachers stimulate higher-order thinking once a month to once a week, where they focus mostly on encouraging students to engage in higher-order thinking.

#### Profile 2

Teachers in profile 2 believe that it is important to use new technology, and that it can be a useful tool to enrich their teaching. However, these teachers think that it is not easy to use new technology, feel dependent on context factors such as technical support and do not feel very capable in using new technology. Also, they do not feel encouraged or discouraged by their colleagues and school director to use new technology. These teachers use new technology approximately a few times a year.

Furthermore, teachers in profile 2 think that stimulating higher-order thinking is important, but less strongly than teachers with profile 1. However, they are unsure whether higher-order thinking is suitable for ‘weak’ learners, feel somewhat dependent on context factors and feel somewhat capable in stimulating higher-order thinking. These teachers stimulate higher-order thinking approximately a few times a year to a few times a month, where they focus mostly on encouraging students to engage in higher-order thinking.

#### Profile 3

Teachers in profile 3 believe that it is important to use new technology, and that it can be a useful tool to enrich their teaching. These teachers think that using new technology is not very difficult, feel somewhat dependent on context factors and do not feel very capable in using new technology. These teachers perceive little support from their colleagues and school director to use new technology and use new technology a bit less than a few times a year.

Furthermore, teachers in profile 3 do not think that stimulating higher-order thinking is very important and do not feel very capable in stimulating higher-order thinking. They do not feel dependent upon context factors such as a method and they do not think that higher-order thinking is more appropriate for ‘smart’ students. Teachers with this profile stimulate students’ higher-order thinking significantly less often (a few times a year) than teachers with other profiles do.

#### Teacher characteristics per profile

As can be seen in Table 4, profile 2 is the most common profile. Furthermore, it appears that profile 3 is represented almost completely by pre-service teachers. Further analysis on this profile showed that 83.2% of the pre-service teachers in this profile come from the same teacher education college.

**Table 4:** Characteristics of participants in each cluster

	Profile 1		Profile 2		Profile 3		Total	
	(N = 197)		(N = 330)		(N = 131)			
	*N*	*%*	*N*	*%*	*N*	*%*	*N*	*%*
Gender								
Male	56	28.4	60	18.2	19	14.5	135	20.5
Female	141	71.6	270	81.8	112	85.5	523	79.5
Teacher type								
In-service teacher	100	50.8	153	46.4	4	3.1	257	39.1
Pre-service teacher	97	49.2	177	53.6	**127**	**96.9**	401	60.9

### Stage 2: Understanding teacher profiles

To gain a more in-depth understanding of the identified profiles we conducted follow-up focus group interviews. As described in section 2.4.1., we selected schools and teacher education colleges where a certain profile was strongly represented. However, participants from the teacher education college where profile 3 was strongly represented were unable to participate in the focus group interviews. Therefore, only participants from schools and teacher education colleges where profile 1 and 2 were strongly represented, participated in the interviews.

#### Matching profiles

We evaluated whether teachers’ own choice of profile matched the results from the cluster-analysis. Therefore, we analyzed the answers participants gave in response to the question: Which profile fits you best? Because some participants did not include a code for either the TANT- SHOT or open-ended questionnaire we could only match the results for 14 out of 21 participants. For 13 of the 14 participants, the chosen profile matched the results of the cluster-analysis. This means that if according to the cluster analysis a participant belonged in a certain profile, the teacher also selected that profile as the best fitting profile. Of these 13 participants, 9 belonged to profile 1, and 4 belonged to profile 2.

#### Recognizing profiles

##### Profile 1

We found that 11 in-service teachers and 2 pre-service chose profile 1 because they recognized themselves in a number of characteristics of this profile (see Table 5). The characteristic that they mentioned most was that stimulating HOT is important for *all* students, both weak and smart. Furthermore, teachers mentioned that using new technology in teaching is very important to prepare students for the future. In one of the interviews a participant explained: “… I find it very important that you prepare students for the future, because the future is all about new technologies.”

**Table 5:** Characteristics that participants (N = 13), who chose profile 1 recognized

Quote	*N*
Stimulating HOT is suitable for *all* students	8
Using new technology is very important to prepare students for the future	5
New technology is a useful tool to enrich and stimulate students' learning	4
Stimulating HOT is very important	3
Using new technology in teaching is not very difficult	2
The use of new technology is stimulated by colleagues and school director	2
I do not need a method or ready-made materials to be able to stimulate students' HOT	2
I feel capable in the use of new technology	1
I use technology often in my lessons	1

Next, participants explained which, if any, characteristics they did not recognize (see Table 6). The participants did not recognize the characteristic of profile 1 that no technical support was needed. Five participants indicated that sometimes they do need ICT- or technical support.

**Table 6:** Characteristics that participants (*N* = 13), who chose profile 1, did not recognize

Quote	*N*
I *do (sometimes) need* technical support to be able to use new technology in my lessons	5
I think that the use of new technology *is not* very appreciated by my colleagues	3
I *do not* always feel capable in stimulating HOT in my students	2
Using new technology *is* difficult	1

We asked teachers with profile 1 about their new technology use, because the results from the cluster-analysis showed that even though these teachers are positive about using new technology, their actual use is limited. Six participants explained that they mainly use drill-and-practice (digital flashcards) and quiz-software. They had an interactive whiteboard and tablets available to work with this software.

##### Profile 2

We found that 3 in-service teachers and 5 pre-service teachers recognized themselves mostly in profile 2. Five participants mentioned that they recognized the characteristic ‘not feeling very capable in using new technology in teaching.’ Furthermore, four participants mentioned that they find it important to use new technology to prepare students for their future (see Table 7).

**Table 7:** Characteristics that participants (*N* = 8) who chose profile 2 recognized

Quote	*N*
I do not feel very capable in using new technology in my teaching	5
Using new technology is important to prepare students for the future	4
It is important to stimulate HOT in students	2
New technology can be a useful tool to enrich and stimulate students' learning	1
I need some explanations before I dare to use new technology in my teaching	1
I sometimes need technical support	1
I need a method to stimulate HOT in students	1
I am unsure whether 'weak' students are capable in HOT	1

In response to the question which characteristics they did not recognize, three participants answered that they believed that the use of new technology was encouraged by colleagues and the school director. Furthermore, three participants mentioned that they believe that stimulating HOT is important for both ‘weak’ and ‘smart’ students (see Table 8).

**Table 8:** Characteristics that participants (N = 8), who chose profile 2, did not recognize

Quote	*N*
I believe that new technology use is encouraged by colleagues and the school director	3
I think that stimulating HOT is important for ‘weak’ and ‘smart’ students	3
Colleagues are sometimes hesitant to use new technology, but this might be caused by a lack of knowledge	1
Colleagues don’t always appreciate creative (HOT) lessons	1

## Conclusions and discussion

In this study, we aimed to answer two research questions, namely (a) which teacher profiles can be identified based on teachers’ attitudes towards using new technology in teaching and stimulating higher-order thinking in students? And (b) do teachers recognize the identified profiles?

The work presented in this study expands upon previously conducted research, by exploring teachers’ attitudes towards using new technology and towards stimulating students’ higher-order thinking simultaneously. We believe that it is important to study these attitudes simultaneously (even though they are not necessarily related) because previous research shows that new technology can be used as a tool to engage students in higher-order thinking better, than if such technologies are not used (Araiza-Alba et al., 2021; Chiang et al., 2014; Passig et al., 2016; Tangkui & Keong, 2020). It is important to stimulate students in higher-order thinking, because this may help them become “good” thinkers (Elder, 2003; Schulz & FitzPatrick, 2016). Therefore, we believe that it is valuable to explore teachers’ attitudes towards teaching approaches that may support students in this. Exploring these teacher attitudes may allow us to develop tailored teacher professionalization to support different groups of teachers in their use of new technology to support students’ higher-order thinking.

### Teacher profiles

The results of our cluster-analysis revealed three profiles based on teachers’ attitudes towards using new technology and stimulating students’ higher-order thinking. Furthermore, in follow-up focus group interviews we found that most participants were able to select a profile because they recognized themselves in one of the profiles. Also, the results of the cluster-analysis matched the teachers’ self-chosen profiles in almost all cases. These results indicate that we can suitably characterize teachers based on their attitudes towards using new technology and stimulating higher-order thinking.

We found that teachers, irrespective of their profile, make little use of new technology and/or do not stimulate higher-order thinking in students very often. This is in line with previous research (Backfish et al., 2020; Fraillon et al., 2013; Voogt et al., 2016). The three identified profiles suggest that the reasons for the limited use of new technology and limited stimulation of students’ higher-order thinking may be different for different groups of teachers. This indicates that it might be necessary to tailor teacher professionalization to the needs of different groups of teachers.

#### Profile 1

Teachers in profile 1 can be characterized as teachers with a positive attitude towards using new technology and towards stimulating higher-order thinking. These teachers stimulate students’ higher-order thinking significantly more often than teachers in the other profiles, with a main focus on encouraging students to engage in complex thinking (approximately a few times a month to once a week). However, despite their positive attitudes towards using new technology, teachers in profile 1 still make very little use of new technology in their teaching (approximately a few times a year). This implies that when teachers stimulate pupils’ higher-order thinking, they predominantly do so with teaching materials or assignments that do not include new technology.

When profile 1 teachers use technology, they mainly seem to use common technology to stimulate students’ *lower-*order thinking. During the interviews, teachers mentioned that they use drill-and-practice software and quiz-software to have students remember facts and check whether and how many students know the right answer on a specific question. This focus on using common technology for *lower-*order thinking is in line with other research (O’Neal et al., 2017; Smeets, 2020).

Since teachers in profile 1 have positives attitude towards using new technology and stimulating higher-order thinking, we hypothesize that attitude-focused professionalization may be less important for this group of teachers compared to teachers in other profiles. Instead, support for teachers in profile 1 might be focused on acquiring knowledge and skills about *how* new technology can be used for stimulating students’ higher-order thinking. This might be done by providing examples of new technology use for stimulating students’ higher-order thinking or by letting them plan and execute lessons in which they use new technology to stimulate students’ higher-order thinking.

#### Profile 2

In our sample, most teachers were categorized in profile 2. Teachers in this profile believe new technology is important and that it is a useful tool to enrich their teaching. However, they think that it is difficult to use new technology, feel dependent upon context factors (such as technical support) and do not feel very capable in using new technology, which might explain their limited use of new technology (approximately a few times a year). Furthermore, these teachers believe that it is fairly important to stimulate higher-order thinking in students but are unsure whether this is suitable for low-achieving students and feel dependent upon context-factors (such as ready-made materials) to be able to stimulate higher-order thinking. These teachers engage in teaching activities aimed at stimulating higher-order thinking a bit less than once a month and encourage students to engage in complex thinking approximately a few times a month. Since these teachers make little use of new technology in their teaching, and only occasionally stimulate students’ higher-order thinking, we suspect that these teachers rarely use new technology for stimulating higher-order thinking.

Based on these findings we hypothesize that it is important to focus support for teachers in profile 2 on enhancing their self-efficacy and lowering their feelings of dependency on context factors. Bowman et al. (2020) found that both teachers’ ability beliefs (e.g., self-efficacy) and value beliefs (e.g., attitude and perceived usefulness of technology) impact technology integration aimed at engaging students in both lower-order and higher-order thinking. Teachers in profile 2 already have reasonably high value beliefs, so it seems important to focus on improving their ability beliefs. This might be done by engaging teachers in attitude-focused professionalization. In such professionalization the focus might be on increasing feelings of confidence and raising awareness about teachers own attitudes. For example, teachers in profile 2 might believe that using new technology is ‘just hard’ and ‘I can’t use it’. By explicitly paying attention to such perceptions by asking questions such as: ‘why do you think you can’t use it?’ ‘What does it mean if you can’t?’ ‘What if you could?’ ‘Can you learn how to use it? ‘What would you need for that?’ teachers may become aware of and challenge their perceptions. Such strategies, along with allowing teachers to practice in a safe environment, receiving feedback from an expert on their use of new technology and stimulation of higher-order thinking and providing examples and materials that teachers can use and explore might help them develop more positive attitudes. In the context of science teaching, such attitude-focused professionalization has proven to be successful in increasing feelings of self-efficacy and lowering feelings of context-dependency (Van Aalderen-Smeets & Walma van der Molen 2015) and might be useful in this context as well.

#### Profile 3

Teachers in profile 3 can be characterized as teachers who have a neutral attitude towards using new technology in teaching and a negative attitude towards stimulating higher-order thinking. These teachers do not think that it is very important to stimulate higher-order thinking, do not feel capable in stimulating higher-order thinking, and rarely stimulate students’ higher-order thinking (approximately a few times a year). Furthermore, they do not feel very encouraged by their colleagues and school director to use new technology. Based on the results regarding the limited use of new technology and stimulating higher-order thinking, we suspect that these teachers very rarely use new technology for stimulating students’ higher-order thinking.

In our sample, teachers in profile 3 were almost all pre-service teachers from the same teacher education college. This is interesting, because this suggests that the educational program these pre-service teachers follow might impact their attitudes. Tondeur et al. (2012) identified 12 strategies that need to be in place in teachers’ educational program to prepare future teachers to use technology. Six of these strategies are related to preparing pre-service teachers at the micro-level: (1) using teacher educators as role models, (2) letting students reflect on digital applications in teaching and learning processes, (3) learning how to use technology by design, (4) collaborating with peers, (5) scaffolding authentic knowledge experiences (6) providing ongoing feedback. In a follow-up study, Tondeur et al. (2018) found that when pre-service teachers perceive more of the occurrences of these six themes during their pre-service training, they report higher competencies in using ICT for learning. Furthermore, Tiba and Condy (2021) found that if pre-service teachers can work with technology during workshops as part of the educational program and if there are technological resources available at the teacher education college, this can impact pre-service teachers’ readiness to use technology. Additionally, Baran et al. (2019) found what teacher educators do, for example whether they serve as role model, can impact pre-service teachers’ beliefs about the value of technology integration. This can result in more positive attitudes about technology integration. These findings indicate that a teacher education program and the actions of teacher educators can impact pre-service teachers attitudes and/or use of technology in their teaching.

Although, we were unable to find studies that explore whether pre-service teachers’ attitudes towards stimulating higher-order thinking might be impacted by the educational program and teacher educators, it seems reasonable to think that whether and how much attention is paid to stimulating higher-order thinking in an educational program and to what extent teacher educators encourage pre-service teachers to stimulate higher-order thinking in their students might impact their attitudes towards stimulating higher-order thinking.

To support teachers in profile 3, it seems important to gain a better understanding about why these teachers have a negative attitude towards stimulating higher-order thinking and whether and to what extent this might be related to the educational program these teachers follow. Support might be incorporated in the educational program and/or teacher educators might be supported in how to teach pre-service teachers about the use of new technology and stimulating higher-order thinking.

### Critical reflections and recommendations for future work

As described in the introduction, we had several reasons to measure teachers’ attitudes towards using new technology and stimulating higher-order thinking separately. In addition, we measured teachers self-reported behavior related to their new technology use and practices to stimulate students’ higher-order thinking. Based on our findings we suspect that the teachers with different profiles make little use of new technology *for* stimulating higher-order thinking. However, we did not measure this directly. Therefore, some behavioral scales related to using common technology for both lower- and higher-order thinking and using new technology for stimulating both lower- and higher-order thinking might be included in a future study. By adding such scales, we can gain insight into how often teachers use common and new technology and to what extent they use such technologies for stimulating lower- and higher-order thinking in students.

Although it is promising that the participants in our follow-up focus group interviews found the profiles recognizable and that the results of the cluster-analysis matched the teachers’ self-chosen profiles in almost all cases, we do not have enough data to validate the profiles that we identified. We suggest that researchers in the future validate the profiles we identified in this study by conducting additional interviews. Such interviews might help in gaining a better understanding of *why* teachers fall into these categories.

This study was conducted just before and at the start of the Covid-19 pandemic. The pandemic caused many teachers to switch from face-to-face to online teaching. Although switching to online teaching does not necessarily mean that teachers use new technology to support and enrich students’ learning, it may have affected their perspectives regarding the use of new technology in teaching as well as their perspectives on engaging students in higher-order thinking. However, we do not yet know whether and how this switch to online teaching impacts teachers’ attitudes towards using new technology or stimulating higher-order thinking, and whether this effect is similar for teachers with different profiles. The present study may be replicated to explore possible changes in teachers’ attitudes.

The profiles that we identified in this study are based on both pre- and in-service teachers' attitudes towards new technology use and stimulating students’ higher-order thinking. These teachers are nested within schools and teacher education colleges and research shows that factors such as school culture and available support can impact teachers’ attitudes (e.g., Van der Linde et al., 2014). The finding that teachers in profile 3 are almost all from the same teacher education college seems to indicate that such factors are important to consider when studying these teachers’ attitudes. Furthermore, pre-service teachers generally have less teaching experience, and this can impact teachers’ technology integration (Backfish et al., 2020). It is important to be aware of factors such as (school) culture and teaching experience since this might have an impact on the strategies that are suitable to support both pre- and in-service teachers in the different profiles.

## CRediT

Frances Wijnen: Conceptualization, Methodology, Formal analysis, Investigation, Writing – original draft, Visualization, Project Administration, Funding Acquisition.

Juliette Walma van der Molen: Conceptualization, Methodology, Writing – Review & Editing, Visualization, Supervision, Project Administration, Funding Acquisition.

Joke Voogt: Conceptualization, Methodology, Writing—Review & Editing, Visualization, Supervision, Funding Acquisition.

The datasets analysed during the current study are available from the corresponding author on reasonable request.

## Appendix 1: Profile descriptions handed out to participants during the focus group interviews

### Profile 1: Ellen

Ellen thinks it is very important to use new technology in her teaching to prepare students well for their future. She thinks that new technology is a useful tool to support and enrich her students learning. She thinks that using new technology is not very difficult and feels reasonably capable in using new technology in her lessons. To do this, she does not necessarily require technical assistance or content support from an ICT-coordinator. Furthermore, she has the feeling that her colleagues and school director appreciate the use of new technology. Ellen uses new technology approximately a few times a year in her lessons.

In addition, Ellen thinks it is very important to stimulate students’ higher-order thinking. She thinks that this is important for *all* students, both ‘strong’ and ‘weak’ students. She feels reasonably capable in stimulating higher-order thinking in her students and does not necessarily need a method or ready-made materials to do this. On average, she pays attention to stimulating students’ higher-order thinking a few times a month.

### Profile 2: Barbara

Barbara thinks it is important to use new technology in her teaching to prepare students well for their future. She also thinks that new technology can be a useful tool to support and enrich her students learning, but she also thinks that is sometimes pretty difficult to use new technology and she needs technical support and help from the ICT-coordinator to do this. She does not feel very capable to use new technology in her lessons. Furthermore, she has the feeling that her colleagues and school director are quite neutral about the use of new technology, it is not disapproved but also not encouraged. Barbara uses new technology approximately a few times a year in her lessons.

In addition, Barbara thinks it is important to stimulate students’ higher-order thinking. She feels reasonably capable to do this but is unsure whether higher-order thinking can also be stimulated in ‘weak’ students. She is also in need of a method and ready-made materials that she can use to stimulate higher-order thinking in students. On average, she pays attention to stimulating students’ higher-order thinking once a month.

### Profile 3: Tineke

Tineke thinks that is reasonably important to use new technology in her teaching to prepare students well for their future. She also thinks that new technology can be a useful tool to support and enrich her students learning. She thinks that it can be difficult to use new technology and appreciates technical support or help from the ICT-coordinator, but it is not a condition. She does not feel very capable in the use of new technology in her lessons. Furthermore, she does *not* have the feeling that her colleagues or the school director believe that it is important to use new technology in teaching. Tineke uses new technology approximately a few times a year in her lessons.

In contrast to the use of new technology, Tineke does not think that it is important to stimulate students’ higher-order thinking. Also, she does not feel capable to stimulate her students’ in higher-order thinking, but she also does not need a method or ready-made materials that she can use stimulate students’ higher-order thinking. However, she does think that higher-order thinking can be stimulated in both ‘strong’ and ‘weak’ students. On average, Tineke stimulates her students to engage in higher-order thinking once a year.
